# Limited genetic diversity and high differentiation in *Angelica dahurica* resulted from domestication: insights to breeding and conservation

**DOI:** 10.1186/s12870-022-03545-5

**Published:** 2022-03-24

**Authors:** Rong Huang, Yinrong Liu, Jianling Chen, Zuyu Lu, Jiajia Wang, Wei He, Zhi Chao, Enwei Tian

**Affiliations:** 1 School of Traditional Chinese Medicine, Southern Medical University, Guangzhou, 510515 China; 2 Department of Pharmacy, Zhujiang Hospital, Southern Medical University, Guangzhou, 510282 China; 3 Guangdong Provincial Key Laboratory of Chinese Medicine Pharmaceutics, Guangzhou, 510515 China; 4 Guangdong Provincial Engineering Laboratory of Chinese Medicine Preparation Technology, Guangzhou, 510515 China

**Keywords:** Genetic diversity, Genetic differentiation, SSR, Cluster analysis, *Angelica dahurica*, Domestication

## Abstract

**Background:**

*Angelica dahurica* belongs to the Apiaceae family, whose dry root is a famous traditional Chinese medicine named as “Bai zhi”. There are two cultivars (*A. dahurica* cv. ‘Hangbaizhi’ and *A. dahurica* cv. ‘Qibaizhi’), which have been domesticated for thousands of years. Long term artificial selection has led to great changes in root phenotypes of the two cultivars, and also decreased their adaptability to environment. We proposed hypothesis that the cultivars may have lost some of the genetic diversity found in the wild species and may be highly differentiated from the latter during the domestication process. However, few studies have been carried out on how domestication affected the genetic variation of this species. Here, we accessed the levels of genetic variation and differentiation within and between wild *A. dahurica* populations and two cultivars using 12 microsatellite markers.

**Results:**

The results revealed that the genetic diversity of the cultivars was much lower than that of wild *A. dahurica*, and *A. dahurica* cv. ‘Qibaizhi’ had lower genetic diversity compared to *A. dahurica* cv. ‘Hangbaizhi’. AMOVA analysis showed significant genetic differentiation between the wild and cultivated *A. dahurica* populations, and between *A. dahurica* cv. ‘Hangbaizhi’ and *A. dahurica* cv. ‘Qibaizhi’. Results from Bayesian, UPGMA, NJ and PcoA clustering analysis indicated that all 15 populations were assigned to two genetic clusters corresponding to the wild and cultivated populations. Bayesian clustering analysis further divided the cultivated populations into two sub-clusters corresponding to the two cultivars.

**Conclusions:**

Our study suggests that the domestication process is likely the major factor resulting in the loss of genetic diversity in cultivated *A. dahurica* populations and in significant genetic differentiation from the wild populations due to founder effect and/or artificially directional selections. This large-scale analysis of population genetics could provide valuable information for genetic resources conservation and breeding programs of *Angelica dahurica*.

## Background

Medicinal plants have been used as important sources of medicine to prevent and treat many human diseases in traditional cultures all over the world for thousands of years [1, 2]. However, given the dramatically increasing demand for plant-derived medicine, many medicinal plants are under over-exploitation, increasing their risk of extinction in the wild [3, 4]. Generally, wild resource contains great genetic variations and beneficial genes before domestication and artificial selection, providing a reservoir of genetic variation for exploiting in breeding efforts [2]. Therefore, effective measures for the conservation need to be taken to further protect the wild resources of medicinal plants. Genetic diversity underlies adaptation and evolution of plants, which allows for dealing with various biotic and abiotic stresses in changing environments [5, 6]. It is also the basis of the plasticity of secondary metabolism, and thus the production of medicinal compounds [7, 8]. Therefore, investigating the genetic variation of medicinal plants in the wild is vital to plan conservation strategies for preserving medicinal plants, as well as breeding programs [9, 10].

Plant domestication is an episode in which human-mediated selection favors phenotypic modification of wild resources to meet human needs, mainly through artificial selection [11, 12]. Domestication of plants not only modifies their phenotypes but also has major impacts on the genetic variation [13–15]. One common genetic effect of domestication is the decrease of genetic diversity compared to their wild resources [16]. The loss of genetic diversity may lead to the reduction of the ability to long-term survival and evolution in changeable environments [17, 18]. It is reported that the extent of the loss of genetic diversity may differ considerably among domesticated plants due to various life-history traits and evolutionary history [19]. For example, about one-third of genetic diversity was lost in soybean [20] and maize [21] compared to their wild relatives, while a majority of genetic diversity was lost in wheat [22]. The difference in extent of the loss of variation lies on the initial population size and the duration of that period [13]. Thus, knowledge of how domestication affects genetic diversity and structure across the range of both wild and cultivated populations is critical for the management and improvement of cultivars of medicinal plants in the future [15, 23]. Up to date, most studies on the effect of domestication on genetic diversity have focused on agronomic crops [12–14, 19, 24], with less attention being paid to medicinal crops. Although a small number of all known medicinal plants have been studied on their population genetics, e. g. *Scrophularia ningpoensis* [8], *Atractylodes macrocephala* [25] and *Cannabis species* [24], numerous ongoing domestication processes have yet to be studied, and these have been of interest to medicinal plant genetic resources conservation and breeding programs.

*Angelica dahurica* (Hoffm.) Benth. et Hook. f. ex Franch. & Sav., is a perennial herb belonging to the genus *Angelica* of the family Apiaceae, mainly distributed in North and Northeast China, Japan, Korea, Russia (Siberia) [26]. Dichogamy has been detected in *A. dahurica*, which is an effective mechanism to encourage outcrossing and avoid selfing [27]. The pollination mechanisms of the Umbelliferae plants were always thought to be wind or insect pollination [28]. Such mechanisms have been observed in the *Angelica* species, such as *A. biserrata* [29]. Therefore, the mating system of *A. dahurica* is considered to be outcrossing. The dry root of the species, named as “bai zhi” (Angelicae Dahuricae Radix), is a famous traditional Chinese medicine, which has been used as a food additive as well as a folk medicinal therapy of headache, rhinitis, cold and toothache amongst others in East Asian countries (Korea, China, and Japan) for thousands of years [30–32]. Two cultivars, *A. dahurica* cv. ‘Hangbaizhi’ and *A. dahurica* cv. ‘Qibaizhi’, widely cultivated in China, have gone through evolution by artificial selection for more than 1000 and 200–400 years, respectively [26, 30, 33]. Long term artificial selection has led to great changes in the root phenotypes of the two cultivars compared with their wild species, and also decreased the adaptability (disease and insect resistances) to environment [26, 34]. Herein, we supposed that the cultivated *A. dahurica* (the two cultivars) may have lost some of the genetic diversity found in the wild species and may be highly differentiated from the latter during the domestication process in the past decades. However, so far, few studies have concerned on how domestication affects the genetic variation of cultivars of *A. dahurica*. There is still lack of studies on genetic diversity and population structure of *A. dahurica* and it culitvars.

In a previous study, we developed highly polymorphic SSR markers by transcriptome sequencing for *A. dahurica* [35], providing efficient molecular markers to conduct population genetics on *A. dahurica*. In this study, we aim to: (1) access the levels of genetic variation and differentiation within and between wild *A. dahurica* and its cultivars; (2) clarify how the domestication process influence on genetic variation of this species. Hopefully, the outcomes of this study could provide valuable information for genetic resource conservation and breeding programs of *A. dahurica*.

## Results

### Allele frequency distribution patterns and genetic diversity

**Fig. 1:**
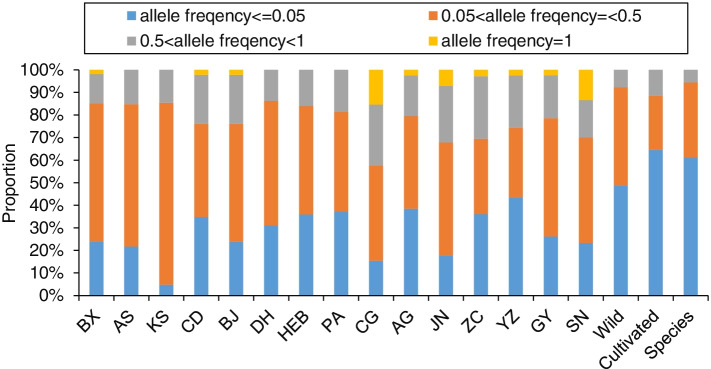
Distribution of allele frequency in populations of *Angelica dahurica* and its two cultivar, *A. dahurica* cv. ‘Hangbaizhi’ and cv. ‘Qibaizhi’

A total of 336 individuals from 15 populations were genotyped with 12 SSR markers, resulted in 108 alleles, of which 90 and 80 were detected in wild and cultivated populations, respectively (Table 1). On average, 9.0 ± 2.7 (range 6–16) alleles per locus were observed. There were 24 private alleles in 11 populations, including 13 alleles in six wild populations and 11 alleles in five cultivated populations (Table 1). The allele frequency distribution patterns are shown in Fig. 1. At species level, rare allele (allele frequency ≤ 5%) accounted for the highest proportion (61.11%). Medium-to-high frequency allele (50% < allele frequency < 100%) were much less than low-to-medium frequency allele (5% < allele frequency ≤ 50%) (5.56% vs 33.33%). There was no common allele (i.e. found in all individuals within *A. dahurica*, allele frequency = 100%) in *A. dahurica*. Wild and cultivated *A. dahurica* displayed the same allele frequency distribution patterns. Among all populations except for two cultivated populations (ZC and YZ), low-to-medium frequency allele accounted for the highest proportion (41.30–80.49%), followed by rare allele (4.88–38.46%) and medium-to-high frequency allele (12.96–25.00%). Common alleles were the least prevalent one (0–13.33%).

**Table 1:** Summary of genetic diversity analysis for 15 populations of *Angelica dahurica* assessed with 12 microsatellite loci

Pop.	Sample size	Alleles	*A*p	*N*a	*N*e	*H*o	*H*e	*I*	*F*_IS_
Wild (*Angelica dahurica*)									
BX	24	54	2	4.500	2.469	0.573	0.537	1.039	−0.130
AS	17	46	1	3.833	2.403	0.590	0.529	0.948	−0.100
KS	24	41	0	3.417	2.351	0.558	0.532	0.947	−0.051
CD	24	46	3	3.833	1.922	0.463	0.403	0.760	−0.121
BJ	24	46	2	3.833	1.966	0.403	0.430	0.810	0.152^**^
DH	14	58	3	4.833	2.818	0.605	0.563	1.110	−0.004
HEB	20	50	2	4.167	2.464	0.470	0.474	0.915	0.090
Mean	21	49	1.9	4.059	2.342	0.523	0.495	0.933	
Cultivars									
*Angelica dahurica* cv. ‘Hangbaizhi ‘									
PA	24	43	4	3.583	1.922	0.420	0.395	0.723	−0.011
GY	24	42	2	3.500	1.968	0.367	0.384	0.730	−0.005
SN	24	30	0	2.500	1.678	0.232	0.265	0.475	0.372^**^
Mean	24	38.3	2	3.194	1.856	0.340	0.348	0.643	
*Angelica dahurica* cv. Qibaizhi									
AG	24	39	3	3.250	1.835	0.346	0.340	0.621	0.222^**^
CG	21	26	0	2.167	1.511	0.329	0.264	0.426	−0.262
YZ	24	39	1	3.250	1.623	0.299	0.307	0.567	0.188^**^
JN	24	28	0	2.333	1.511	0.249	0.250	0.437	0.118
ZC	24	36	1	3.000	1.578	0.281	0.288	0.521	0.059
Mean	23.4	33.6	1	2.800	1.612	0.301	0.290	0.514	
Cultivars Mean	23.6	35	1.4	2.948	1.703	0.316	0.312	0.562	
Species Mean	22.4	42	1.6	3.467	2.001	0.412	0.397	0.735	

Note: *A*p: number of private alleles; *N*a: number of observed alleles; *N*e: number of effective alleles; *I*: Shannon’sindex; Ho: observed heterozygosity; *H*e: expected heterozygosity; *F*_IS_: inbreeding coefficient

^**^p < 0.01

At species level, the average values of observed heterozygosity (*H*o) and Shannon’s index (*I*) were 0.412 and 0.735, respectively (Table 1). The wild *A. dahurica* had, on average, significantly higher estimates of genetic diversity than did the cultivated one (*H*o: 0.523 ± 0.077 vs 0.316 ± 0.063, *p* = 0.00; *I*: 0.933 ± 0.121 vs 0.562 ± 0.120, *p* = 0.00) (Table 1). Compared to populations of *A. dahurica* cv. ‘Haibaizhi’, populations of *A. dahurica* cv. ‘Qinaizhi’ showed slightly lower genetic diversity (*H*o: 0.301 ± 0.038 vs 0.340 ± 0.097, *p* = 0.08; *I*: 0.514 ± 0.083 vs 0.643 ± 0.145, *p* = 0.19). For each population analyzed, genetic diversity parameters varied widely among populations (Table 1). The highest level of genetic diversity was found in the wild population DH (*H*o = 0.605, *I* = 1.110), and the lowest in the cultivated population JN (*H*o = 0.249, *I* = 0.437). Four populations, including one wild population (BJ) and three cultivated populations (SN, AG and YZ), had significantly positive *F*_IS_ values (Table 1), suggesting their heterozygote deficiency. The remainder showed negative *F*_IS_ values, indicating they presented an excess of heterozygotes.

### Divergence between wild and cultivated *Angelica dahurica*, and between two cultivars

Nonhierarchical AMOVA indicated that 34.53% (*F*st = 0.345) of the total variation was partitioned among populations of *A. dahurica* (Table 2). That is, of total genetic variation, most (65.47%) was due to individual differences within populations (Table 2). Only 19.41% of total variation resided among populations in wild *A. dahurica*, but variation distributed among populations and individuals were almost equal (48.58% vs 51.42%) in cultivated *A. dahurica* (Table 2). That is, population differentiation in cultivated *A. dahurica* is much stronger than that in wild *A. dahurica* (*F*st: 0.486 vs 0.194) (Table 2).

**Table 2:** Analyses of molecular variance (AMOVAs) for 15 *Angelica dahurica* populations assessed with 12 microsatellite loci

Source	d.f.	Variance component	Percentage of variation	*F*-statistics	*p*-value
Nonhierarchical					
Species					
Among populations	14	0.803	34.53%	*F*st = 0.345	0.000
Within populations	657	1.522	65.47%		
Wild					
Among populations	6	0.618	19.41%	*F*st = 0.194	0.000
Within populations	287	2.564	80.59%		
Cultivars					
Among populations	7	0.467	48.58%	*F*st = 0.486	0.000
Within populations	370	0.494	51.42%		
Hierarchical					
Wild vs Cultivated					
Between groups	1	0.719	26.99%	*F*ct = 0.270	0.000
Among populations within group	13	0.423	15.89%	*F*st = 0.429	0.000
Within populations	657	1.522	57.13%		
Haibaizhi vs Qibaizhi					
Between groups	1	0.145	14.78%	*F*ct = 0.148	0.000
Among populations within group	187	0.351	35.84%	*F*st = 0.421	0.000
Within populations	189	0.484	49.38%		

Note: *d. f.* degree of freedom, *Fct* genetic differentiation between groups, *Fst* genetic differentiation among populations within group

Hierarchical AMOVA revealed that 26.99% of the total variation was distributed between wild and cultivated *A. dahurica*, with 15.89 and 57.13% explained by variation among and within populations, respectively (Table 2). In the two cultivars, 14.78% of the total variation was found between *A. dahurica* cv. ‘Hangbaizhi’ and *A. dahurica* cv. ‘Qibaizhi’ (Table 2). Significant genetic divergence was found both between wild and cultivated *A. dahurica* (*F*ct = 0.270, *p* = 0.00), and between *A. dahurica* cv. ‘Hangbaizhi’ and *A. dahurica* cv. ‘Qibaizhi’ (*F*ct = 0.148, *p* = 0.00) (Table 2).

### Genetic structure

**Fig. 2:**
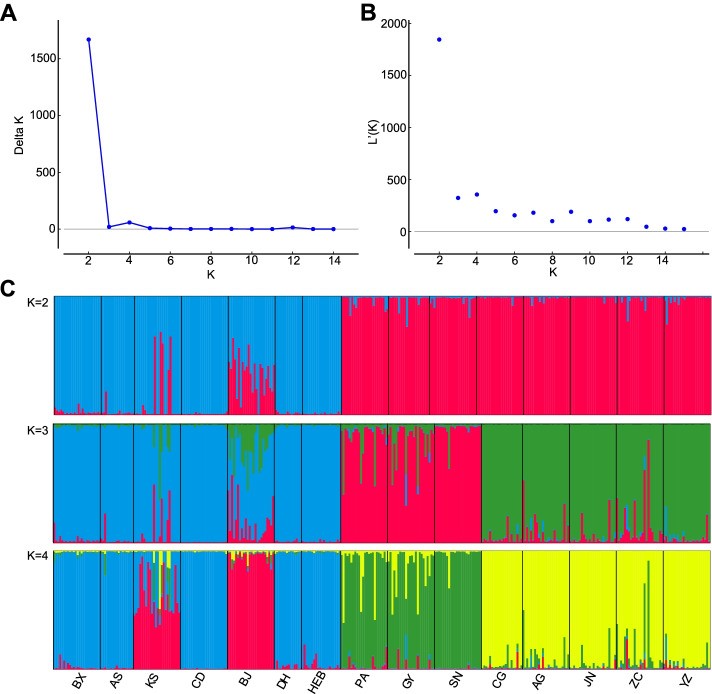
Bayesian clustering results of the STRUCTURE analysis for *Angelica dahurica* and its two cultivar, *A. dahurica* cv. ‘Hangbaizhi’ and cv. ‘Qibaizhi’ assessed with 12 microsatellite loci. **A** Estimates of ΔK with respect to K; **B** Plot of the probability of the data (LnP(D)) values; **C** Genetic group structure with K = 2, 3 and 4

Genetic admixture analysis performed by STRUCTURE revealed that both the maximum value of delta K and the highest log likelihood were observed at K = 2 (Fig. 2A-B), indicating that all populations were assigned to two genetic clusters (Fig. 2C). All individuals in wild populations were assigned to the same genetic cluster, while all individuals in the cultivated populations were assigned to a second genetic cluster. There was a high degree of admixture of two gene pools in most individuals of BJ population and some individuals of KS population. With K = 3, substructure appeared in the cultivated populations, which were divided into two genetic cluster corresponding to the two cultivars (*A. dahurica* cv. ‘Hangbaizhi’ and *A. dahurica* cv. ‘Qibaizhi’), whereas the wild populations remained relatively uniform (Fig. 2C). When K = 4, substructure appeared in the wild populations. All individuals of BJ and KS populations were assigned into the same genetic cluster, with some degree of admixture of three gene pools in most individuals (Fig. 2C).

**Fig. 3:**
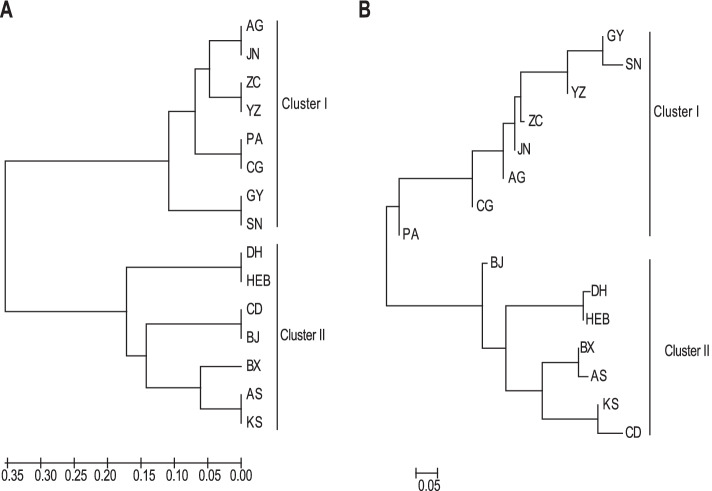
UPGMA dendrogram (**A**) and unrooted neighbour-joining (**B**) tree of seven wild *Angelica dahurica* populations and eight cultivated populations assessed with 12 microsatellite loci

**Fig. 4:**
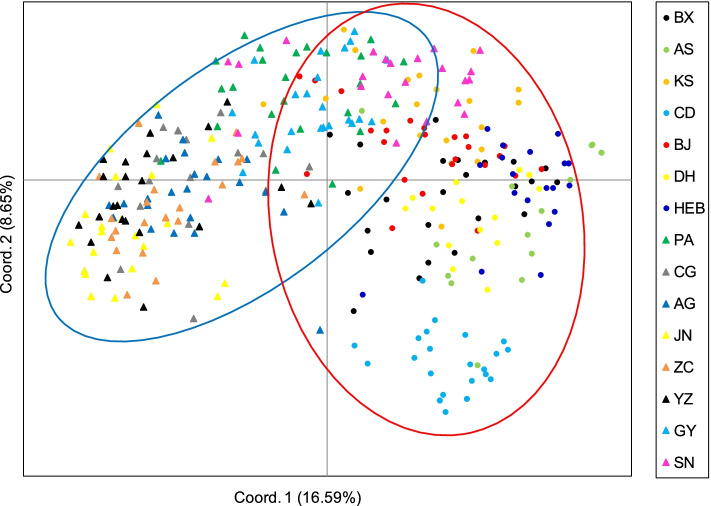
Principal coordinate analysis of 147 individuals from seven wild *Angelica dahurica* populations (circles) and 189 individuals from eight cultivated populations (triangles), assessed with 12 microsatellite loci

The UPGMA dendrogram (Fig. 3A) was broadly consistent with the unrooted neighbor-joining (NJ) tree (Fig. 3B). Fifteen populations were classified into two clusters (I, II), which corresponded to cultivated and wild *A. dahurica*, respectively. PCoA (Fig. 4) largely confirmed the partitioning results of the UPGMA dendrogram, the NJ tree and STRUCTURE analysis, and showed some mixture of populations in both wild (BJ, BX and KS) and cultivated *A. dahurica*.

## Discussion

### Genetic diversity of wild *A. dahurica*

Wild progenitors are always an important genetic resource for plant breeding [36]. However, genetic diversity and population genetic structure of wild populations of *A. dahurica* is poorly understood. In this study, we therefore revealed the genetic diversity and population genetic structure of this species, providing important basic information for the further conservation and breeding effort. The breeding system is always considered to have a major effect on genetic diversity of plant species [6, 37, 38]. Many studies have shown that outcrossing plants tend to maintain a higher genetic diversity compared to selfing plants [39–41]. Our results revealed that genetic diversity of *A. dahurica* was higher than that of selfing species summarized by previous meta-analyses [37, 42]. As an Umbelliferae and dichogamous plant, *A. dahurica* may cross-pollinate by wind or insects [28, 29], which will promote gene exchange between individuals, thus preventing loss of diversity caused by genetic drift. This can be evident by the absence of inbreeding signs in most of wild *A. dahurica* populations. Moreover, the common allele is extremely rare, while the low-to-medium frequency allele is the most prevalent within *A. dahurica* populations, indicating that the populations are heterogeneous. In addition, *A. dahurica* harbors greater genetic diversity within population and a low genetic differentiation among populations as many outcrossing plants [37–42]. However, when compared to other Apiaceae species, with *H*o values ranging from 0.361 to 0.85 [43–47], wild *A. dahurica* showed moderate genetic diversity (*H*o = 0.523). Anthropic activities also have a huge impact on genetic diversity of wild plants as they are usually accompanied with dramatically increasing loss and damage of natural habitats [48, 49]. Theoretically, loss of habitat can cause a decrease of population genetic diversity due to the reduction of population size, increased random genetic drift and inbreeding [6]. During our 6 years (2015–2020) of fieldwork, we observed habitat erosion in *A. dahurica* due to anthropic activities caused by urbanization, agricultural and silvicultural practices. In addition, the wild resources are in decline due to over-exploitation. Thus, over-exploitation of the wild resources and habitat destruction are the most likely factors responsible for the moderate level of genetic diversity in *A. dahurica*. Among the seven wild populations, DH, AS and BX showed relatively high genetic diversity, which could provide abundant genetic variation for breeding. In addition, most of populations preserve private alleles, which are also valuable genetic resources for breeding.

### Genetic consequence of domestication

Many experiential studies have revealed that domestication processes caused a massive loss of genetic diversity in domestication crop plants [12, 50]. However, the extent of the loss of genetic diversity in domesticated medicinal plants have been poorly understood. Here, we assessed this reduction by comparing the levels of genetic diversity between wild and cultivated populations. Our results demonstrated that the average population genetic diversity of cultivated *A. dahurica* was approximately reduced by a third compared to the wild resource, respectively. The findings of our study suggested that domestication had a negative effect on genetic diversity of cultivated *A. dahurica*, and the extent was comparable to many crop plants, e. g. maize and soybean [20, 21].

It has been demonstrated that founder effect or bottleneck effect, selective sweeps and/or directional selection, and intensive breeding are the main factors causing the reduction of genetic diversity [12, 13, 23, 51]. During the initial domestication, the population size is extremely small (founder effect), which will result in enormous decrease of genetic diversity [12]. This might be the cause of *A. dahurica*, which was most likely descended from a limited number of individuals from the wild since more than 1000 years ago [30–32]. Furthermore, directional selection for desirable phenotypes during domestication processes would cause a more drastic loss of diversity [13]. During domestication processes of *A. dahurica*, the phenotype of thick and unbranched root might be continuously selected, resulting in further loss of diversity by the reduction of the effective population size and the increasing genetic drift. Moreover, inbreeding caused further genetic erosion, which is a recent phenomenon caused by the decrease of the efficient population size after domestication [15, 52]. Obvious signs of inbreeding were observed in some cultivated populations (i. e. populations SN, AG and YZ, see Table 1), suggesting that inbreeding may be also partly responsible for the diversity decline.

For the two cultivars, a reduction of genetic diversity from *A. dahurica* cv. ‘Hangbaizhi’ to *A. dahurica* cv. ‘Qibaizhi’ was observed. STRUCTURE cluster analysis showed that the two cultivars clustered into a genetic group, indicating that cultivated *A. dahurica* have possibly originated from the same genetic resource. According to the time of initial cultivation, *A. dahurica* cv. ‘Hangbaizhi’ (more than 1000 years) has a longer domestication history compared to *A. dahurica* cv. ‘Qibaizhi’ (about 200–400 years) [26, 30, 33]. Thus, *A. dahurica* cv. ‘Qibaizhi’ may have originally being introduced from *A. dahurica* cv. ‘Hangbaizhi’. Our result is likely consistent to the fact that genetic diversity decreased along the diffusion history [12, 53, 54]. Unfortunately, as there is no record of domestication origin of the two cultivars, the reason behind this diversity decline is still unclear.

**Fig. 5:**
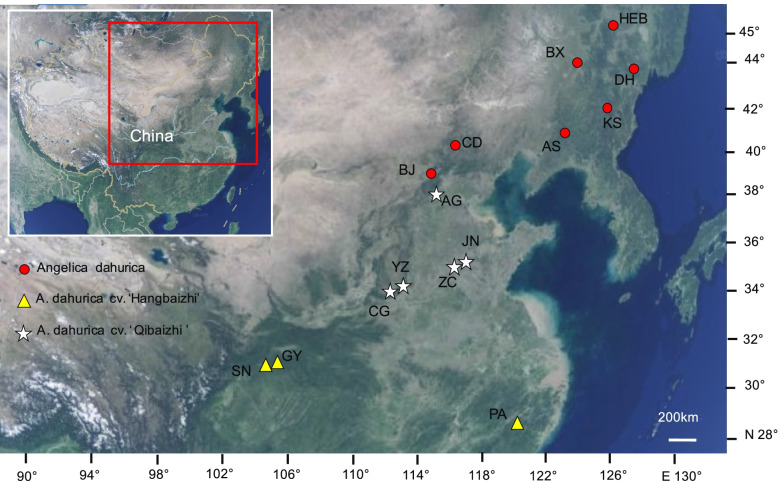
Geographic distribution of sampled populations of *Angelica dahurica* and its two cultivar, *A. dahurica* cv. ‘Hangbaizhi’ and cv. ‘Qibaizhi’. The map was drawn by the authors with reference to Google Maps. The map can be found at https://maps.google.com/

Besides the loss of genetic diversity, genetic divergence between wild plants and their cultivars is another major genetic footprint during plant domestication process [25, 50]. Morphological differences in root had been found between wild and cultivated *A. dahurica*, and between *A. dahurica* cv. ‘Hangbaizhi’ and cv. ‘Qibaizhi’ [26], however, few investigations have been carried out on their genetic divergence. Our results of NJ tree, UPGMA dendrogram and Bayesian genetic structure revealed a distinct divergence between the wild and cultivated *A. dahurica*. PCoA analysis almost confirmed the partitioning results of NJ tree, UPGMA dendrogram and Bayesian genetic structure. Significant genetic differentiation was also detected between the wild and cultivated *A. dahurica* (*F*ct = 0.270, *p* = 0.000) in the AMOVA analysis. Furthermore, the Bayesian genetic structure analysis also indicated an obvious genetic clustering between the two cultivars (*A. dahurica* cv. ‘Hangbaizhi’ and *A. dahurica* cv. ‘Qibaizhi’). Also, AMOVA analysis showed significant genetic differentiation between them (*F*ct = 0.148, *p* = 0.000). Artificial selection as a driving force of divergence between wild and domesticated plants has been documented [55–58]. In this study, the divergence between wild *A. dahurica* and its cultivars are most probably derived from artificial selection of root characteristics during the domestication process. Although the two cultivars possibility originated from the same genetic resource, they are cultivated in different regions since initial cultivation. Therein, *A. dahurica* cv. ‘Hangbaizhi’ was mainly cultivated in Zhejiang and Sichuan Provinces, China, while *A. dahurica* cv. ‘Qibaizhi’ was planted in Henan, Hebei and Anhui Provinces, China (see Fig. 5). Introduction of one cultivar into the growing areas of the other cultivar hardly ever occurred, which may have prevented gene flow between the two cultivars, promoting their genetic differentiation.

### Conservation strategies and utilization in breeding

Our results suggested that the long time artificial selection has resulted in the loss of genetic diversity, and this may lead to the decline of disease and insect resistance in cultivated *A. dahurica*. Generally, wild resources are always recognized as a critical resource for breeding efforts [13]. The significant genetic differentiation observed between wild and cultivated *A. dahurica* (two different gene pools) indicated that the wild resources could be used as a source of novel alleles for improvement of the future genetic improvement of cultivated *A. dahurica*. However, the wild resources are in decline. Based on the knowledge of genetic diversity and structure of *A. dahurica*, an appropriate conservation and management strategy can be formulated here. Conservation of genetic diversity should concentrate on the maintenance of large populations in outcrossing plants due to the fact that most genetic diversity reside within populations [6, 10]. In this case, maintenance of large and diversified populations of *A. dahurica* (i. e. DH, AS and BX) is a key to preserve diversity due to lower differentiation among populations and greater variation within population. These populations with high levels of diversity could provide abundant genetic variation for future breeding program.

## Conclusions

In the present study, we used 12 SSR markers to access the levels of genetic variation and differentiation within and between wild *A. dahurica* and its cultivars from 15 locations. Our results revealed that cultivated *A. dahurica* harbored lower genetic diversity, and showed significantly high genetic differentiation from wild *A. dahurica*. The domestication process through artificial selection is the major factor resulting in the loss of genetic diversity in cultivated *A. dahurica,* considering that significant genetic divergence has been found in the wild resources. Our results could provide genetic insight to improve conservation and management strategies for *A. dahurica*, and advance understanding of effects of domestication on genetic diversity of medicinal plants.

## Methods

### Plant samples collections

Between July 2017 and August 2019, we sampled *Angelica dahurica* wild populations from 7 locations which covered all the species natural distribution area in China. Specimens of *A. dahurica* cv. ‘Hangbaizhi’ and *A. dahurica* cv. ‘Qibaizhi’ were collected from 3 and 5 locations, respectively, which included almost all the main cultivated area (Table 3, Fig. 5). Samples collection protocols are as follow: in each population, the distance between each collected individual plant was over 20 m for both wild and cultivated populations, which aimed to avoid multiple samples from the same clone [35]. We obtained a total of 336 plant individual specimens (fresh leaves) from 15 populations, an average of 22.4 individuals for each population (ranging from 17 to 24 individuals). All the fresh specimens were preserved in gel-dried silica for DNA extraction. For the wild species *A. dahurica* and two cultivars, as so far they have not been listed in national key protected plants, we collected the samples without any required permissions. Associate professor Enwei Tian from the School of Traditional Chinese Medicine, Southern Medical University (SMU), morphologically identified all the voucher specimens mentioned above, which then were deposited in the herbarium of SMU (Table 3). Our field study and Experimental research complied with local legislation, national and international guidelines. The authors also complied with the Convention on the Trade in Endangered Species of Wild Fauna and Flora.

**Table 3:** Summary of sample locations and sample sizes of *Angelica dahurica* and its cultivars

Species	Types	Voucher no.	Locantions (abbreviations)	Latitude, Longitude	Sample sizes
*Angelica dahurica*	Wild	2,017,811-BX(1 ~ 24)	Benxi, Liaoning Province (BX)	44°22′47″ N, 124°57′58″ E	24
	Wild	2,017,808-AS(1 ~ 17)	Anshan, Liaoning Province (AS)	41°00′55″ N, 123°08′05″ E	17
	Wild	2,017,812-KS(1 ~ 24)	Tonghua, Jilin Province (KS)	42°25′49″ N, 126°06′36″ E	24
	Wild	2,017,826-CD(1 ~ 24)	Chengde, Hebei Province (CD)	40°40′12″ N, 117°40′12″ E	24
	Wild	2,018,816-BJ(1 ~ 24)	Beijing (BJ)	39°58′47″ N, 115°25′40″ E	24
	Wild	2,018,824-DH(1 ~ 14)	Dunhua, Jilin Province (DH)	43°34′14″ N, 128°00′50″ E	14
	Wild	2,018,825-HEB(1 ~ 20)	Haerbin, Heilongjiang Province (HEB)	45°42′24″ N, 126°38′37″ E	20
*A. dahurica* cv. ‘Hangbaizhi’	Cultivated	2,018,625-PA(1 ~ 24)	Panan, Zhejiang Province (PA)	28°57′05″ N, 120°28′05″ E	24
	Cultivated	2,019,722-GY(1 ~ 24)	Guangyuan, Sichuan Province (GY)	31°56′38″ N, 105°38′39″ E	24
	Cultivated	2,019,720-SN(1 ~ 24)	Suining,Sichuan Province (SN)	30°34′09″ N, 105°34′49″ E	24
*A. dahurica* cv. ‘Qibaizhi’	Cultivated	2,018,808-YZ(1 ~ 24)	Yuzhou, Henan Province (YZ)	34°12′01″ N, 113°34′32″ E	24
	Cultivated	2,018,808-CG(1 ~ 21)	Changge, Henan Province (CG)	34°11′30″ N, 113°53′45″ E	21
	Cultivated	2,018,814-AG(1 ~ 24)	Anguo, Hebei (AG)	38°25′11″ N, 115°19′37″ E	24
	Cultivated	2,018,810-JN(1 ~ 24)	Jining, Shandong Province (JN)	35°23′11″ N, 116°40′31″ E	24
	Cultivated	2,018,811-ZC(1 ~ 24)	Jining, Shandong Province (ZC)	35°24′36″ N, 116°53′47″ E	24
Total					336

### DNA extraction, SSR-PCR amplification and genotyping

Total genomic DNA was extracted from the dried leaves of each sample using a modified CTAB method [59]. DNA concentration and quality of the exacted DNA were assessed using a NanoDrop 1000 UV/Vis spectrophotometer (Thermo Scientific, Wilmington, DE, USA) and gel electrophoresis in 1.5% agarose, respectively.

Twelve microsatellite markers, previously developed for *A. dahurica* [35], were selected to determine the genetic diversity and population structure of *A. dahurica* and its cultivars. Attributes of the 12 SSR primers are shown in Table 4. The forward primers (5′ end) were labeled with one of the following the forward dyes: TAMRA or FAM. PCR amplification was performed in a volume of 20 μL consisting of 20 ng genomic DNA, 0.2 mM each dNTP, 0.4 μM each primer, 10 × PCR buffer (Mg^2+^ free), 2.5 mM Mg^2+^, 1 unit Taq DNA polymerase (Takara, Dalian, China) with the following procedure: initial denaturation at 95 °C for 5 min, 35 cycles of denaturation at 94 °C for 30 s, annealing at 50 to 55 °C for 60 s, extension at 72 °C for 45 s and a final extension of 72 °C for 8 min. The PCR products were visualized using capillary electrophoresis on an ABI PRISM 3100 Genetic Analyser (Applied Biosystems, Foster City, CA). The size of all alleles was determined on Genotyper 4.0, with LIZ 500 (Applied Biosystems, Foster City, CA) as an internal product size standard.

**Table 4:** Attributes of 12 microsatellite loci used for genotyping of *Angelica dahurica* and its cultivars

Locus	Forward and reverse primer sequences	Motifs	Product size range (bp)	*T*_*a*_	GenBank Accession No.
AD1	F (FAM):TCCTCCAGCTGGCATAATAATAA R:ATTAAAAAGAACAAGGGGCTCAA	(TGC)6	111–123	55	MH220032
AD7	F (FAM):GCTCTCTTAAATTTCACCCCAAC	(ATTACC)4	131–155	55	MH220035
	R:TACTAGATTCTTCCAGAGCGACG				
AD8	F (TAMRA):TTCAACATGGTCATGTGAGTGAT	(GGAGTG)4	140–164	53	MH220036
	R:CCGTTGGAGGTCTTCTTGTAAAT				
AD9	F (TAMRA):CAACACACATGATCCAGAAGAAA R:GAGCTGGAGATAGTCTGTTGCAT	(TCTGCA)10	99–159	50	MH220037
AD10	F (FAM):AGACTGCACCTGTCTCATTTTTC	(GT)9	116–140	50	MH220038
	R:GGCTTGTAATTAATCTTTGCACC				
AD11	F (TAMRA):TTCGTCATTTAGAAACGATAGCA	(TCT)7	127–142	50	MH220039
	R:TCAATGGATACCACCACATCATA				
AD14	F (FAM):TGTACTCCATGGACTGGAGTCTT	(TCA)7	108–123	50	MH220041
	R:TTTGTTTTCTGACAAAGCCAAAT				
AD17	F (TAMRA):GGATCATGTTGATGATGGAAAAT	(AGA)7	145–163	50	MH220042
	R:TTCGATTACTACAGCAGATGAGC				
AD19	F (TAMRA):CCCCATTTCTCCCATAGATAGAT	(GCA)6	125–140	53	MH220043
	R:CCATTAATTGTTCTGCATTTTCC				
AD22	F (FAM): AAACAATATCAAATCAAATGGCG	(TTC)6	84–99	50	MH844986
	R: GTGGTGATGATGAATCTTGTGAA				
AD23	F (FAM): GCTTGACATATATCATCGCCTTT	(CTT)_6_	130–142	50	MH844987
	R:TAGACCAAGAGCCAAATAAACCA				
AD24	F (TAMRA):GCGAGATGGAAATGACAAATTCT	(GCA)_6_	108–117	50	MH844988
	R: ATCCCACCATTTCCTCATTAAGT				

Note: Each forward (5′ end) primer was labeled with TAMRA or FAM fluorescent dyes

### Data analysis

To explore the level of genetic diversity, number of observed alleles (*N*a), effective alleles (*N*e) and private alleles (*A*p), Shannon’s information index (*I*), observed heterozygosity (*H*o), expected heterozygosity (*H*e) over loci were calculated using GenAlEx v6.502 [60]. GenAlEx was also used to calculate allele frequency. Wright’s *F*-statistics inbreeding coefficient (*F*_IS_) [61] was calculated using ARLEQUIN v3.5 [62], with significance determined by permutation (1000 replicates). Subsequently, an analysis of molecular variance (AMOVA) was also implemented in ARLEQUIN to quantify the partitioning of genetic variation and coefficient of genetic differentiation with 999 permutations used for tests of significance.

To understand the genetic relationship among all populations, Bayesian cluster analysis was conducted in the program STRUCTURE v2.1 [63] to assign a certain individual to K genetic clusters. K was set as 1 to 15. Five runs for each K were performed under the admixture model, with a burn-in length of 100,000 and a run length of 1,000,000 Markov chain Monte Carlo (MCMC) replications. The optimal value of K was determined by Evanno test using Structure Harvester [64]. Nei’s genetic distances between populations were calculated in GenAlEx, and used as input for a cluster analysis using both the unweighted pair-group method of arithmetic averages (UPGMA) and neighbour-joining (NJ) method using MEGA X [65], respectively. Principal coordinate analysis (PCoA), as an alternative mean, was performed in GenAlEx to detect and visualize the genetic relationship among populations.

## Data Availability

Raw sequence information are available in National Center for Biotechnology Information (NCBI) Sequence Read Archive (RNA sequence information, SRA: SRP162120 BioProject ID PRJNA490770). Sequence information for the developed EST-SSR primer pairs has been deposited at GenBank (Accession numbers are provided in Table 4). The other datasets supporting the findings of this study are available in the Dryad Digital Repository (10.5061/dryad.d51c5b050).

## Abbreviations

SSR: Simple Sequence Repeat
*N*a: Number of observed alleles
*N*e: Effective alleles
*A*p: Private alleles
*I*: Shannon’s information index
*H*o: Observed heterozygosity
*H*e: Expected heterozygosity
*F*_IS_: Inbreeding coefficient
MCMC: Markov chain Monte Carlo
UPGMA: Unweighted pair-group method of arithmetic averages
NJ: Neighbour-joining method
PCoA: Principal coordinate analysis
d.f.: Degree of freedom
*F*_ct_: Genetic differentiation between groups
*F*st: Genetic differentiation among populations within group
